# Primary care disease management for venous leg ulceration—study protocol for the Ulcus Cruris Care [UCC] randomized controlled trial (DRKS00026126)

**DOI:** 10.1186/s13063-021-05944-9

**Published:** 2022-01-20

**Authors:** Jonas D. Senft, Thomas Fleischhauer, Jona Frasch, Wiebke van Rees, Manuel Feißt, Simon Schwill, Christine Fink, Regina Poß-Doering, Michel Wensing, Uwe Müller-Bühl, Joachim Szecsenyi

**Affiliations:** 1 Department of General Practice and Health Services Research, University Hospital Heidelberg, Im Neuenheimer Feld 130.3, 69120 Heidelberg, Germany; 2 aQua Institute for Applied Quality Promotion and Research in Health Care GmbH, Maschmühlenweg 8–10, 37073 Göttingen, Germany; 3 Institute of Medical Biometry, University of Heidelberg, Im Neuenheimer Feld 305, 69120 Heidelberg, Germany; 4 Department of Dermatology, University Hospital Heidelberg, Im Neuenheimer Feld 440, 69120 Heidelberg, Germany

**Keywords:** Venous leg ulcer, General medicine, Disease management, Randomized controlled trial, Study protocol

## Abstract

**Background:**

Venous leg ulcers (VLU) have a prevalence of 1–2% in developed countries, and affected patients are severely and long-term impaired in daily activities, work, and social participation. Evidence-based outpatient treatment based on compression therapy is frequently not implemented. The “Ulcus Cruris Care” project was established to develop a disease management concept to improve outpatient treatment for patients with VLU in German primary care. For this purpose, a multifaceted intervention was conceived consisting of an online training for general practitioners and medical assistants, standardized treatment recommendations, e-learning and print-based information for patients, and a software support for case management. The main aims of the Ulcus Cruris Care intervention are to promote standardized treatment according to current scientific knowledge, to facilitate case management for VLU patients exerted by medical assistants, and to support patient education and participation in the treatment process. The UCC trial was designed to evaluate the effectiveness of the Ulcus Cruris Care intervention.

**Methods:**

The UCC trial is a prospective cluster-randomized controlled multicenter trial. Fifty GP practices are intended to be recruited and randomized 1:1 to intervention or control arm. Patients with venous leg ulcers will be recruited by participating GP practices, to include a total of 63 patients in each arm. The primary outcome is time to ulcer healing. Secondary outcomes comprise number and sizes of ulcers, recurrence, pain intensity according to the visual analog scale, health-related quality of life according to EQ-5D-5L, depressiveness according to Patient Health Questionnaire (PHQ-9), patient satisfaction according to the Patient Assessment of Chronic Illness Care (PACIC-5A) query, and adherence to VLU treatment. The outcome analysis of the UCC trial is accompanied by a health economic analysis and a process evaluation.

**Discussion:**

The UCC trial will evaluate whether the Ulcus Cruris Care intervention may lead to faster wound healing, higher health-related quality of life, and lower use of medical resources. If the intervention turns out to have a positive impact on assessed outcomes, comprehensive implementation in primary care may be considered.

**Trial registration:**

The trial protocol (version 1 as of July 19, 2021) has been registered in the German Clinical Trials Register on August 30, 2021 (DRKS00026126).

**Supplementary Information:**

The online version contains supplementary material available at 10.1186/s13063-021-05944-9.

## Background

Venous leg ulcers (VLUs) have a prevalence of 1–2% of the population in developed countries and account for up to 70% of chronic leg ulcerations [1, 2]. Even with the best treatment, wound healing may take several months, and affected patients are severely impaired in daily activities, work, and social participation resulting in a reduced quality of life and a high prevalence of psychological disorders [3–6].

Compression therapy counteracts venous hypertension as the underlying pathophysiologic cycle of chronic VLU and has been proven to be highly efficient displaying 2 to 4 times higher healing rates after 12 weeks compared to wound therapy not routinely based on compression [7, 8]. Although compression therapy represents the pillar of evidence-based therapy, it is not applied in a significant proportion of affected patients. Care analyses in Europe show that only 30 to 50% of patients with VLU receive compression therapy [9, 10]. In addition, if applied, devices and modalities of compression are often not chosen adequately to facilitate an exerted pressure of 30–40 mmHg needed for efficient support of venous reflux [11].

As a consequence, affected patients might have to endure prolonged healing processes. Shortcomings in VLU treatment are presumably of multifactorial nature. In recent years, emerging wound dressing technologies strongly shifted the focus of chronic wound treatment to local wound therapy, although there is no firm evidence for a superior wound dressing for VLU [12]. Insufficient knowledge about compression devices and practical application on caregiver-side has been indicated by observational studies [13, 14]. On the other hand, treatment strategies focusing on local wound therapy may promote that patients tend to assume a rather passive role. This is reflected by lacking patient-sided knowledge about VLU therapy [15]. Since patients may have difficulties to tolerate and handle compression devices, passive role and lacking knowledge may compromise adherence.

The “Ulcus Cruris Care” project was established to develop and evaluate an evidence-based and patient-centered disease management concept to improve outpatient treatment for VLU patients in German primary care. According to known disease management concepts, such as for diabetes mellitus or heart failure, the “Ulcus Cruris Care” project aims to establish a disease-specific standardized case management for patients with VLU in general practices. For implementation into practice, a multifaceted intervention was developed consisting of a training for general practitioners (GPs) and medical assistants, standardized treatment recommendations, e-learning and print-based information for patients, and a software tool supporting wound documentation and case management. The main aims of the Ulcus Cruris Care intervention are to promote standardized treatment of VLU according to current scientific knowledge, to facilitate case management for VLU patients exerted by medical assistants, and to support patient education and active participation in the treatment process.

The UCC trial is intended to evaluate the effectiveness of the “Ulcus Cruris Care” intervention for the treatment of patients with VLU compared to usual care in German primary care practices.

## Methods

### Trial design

**Fig. 1:**
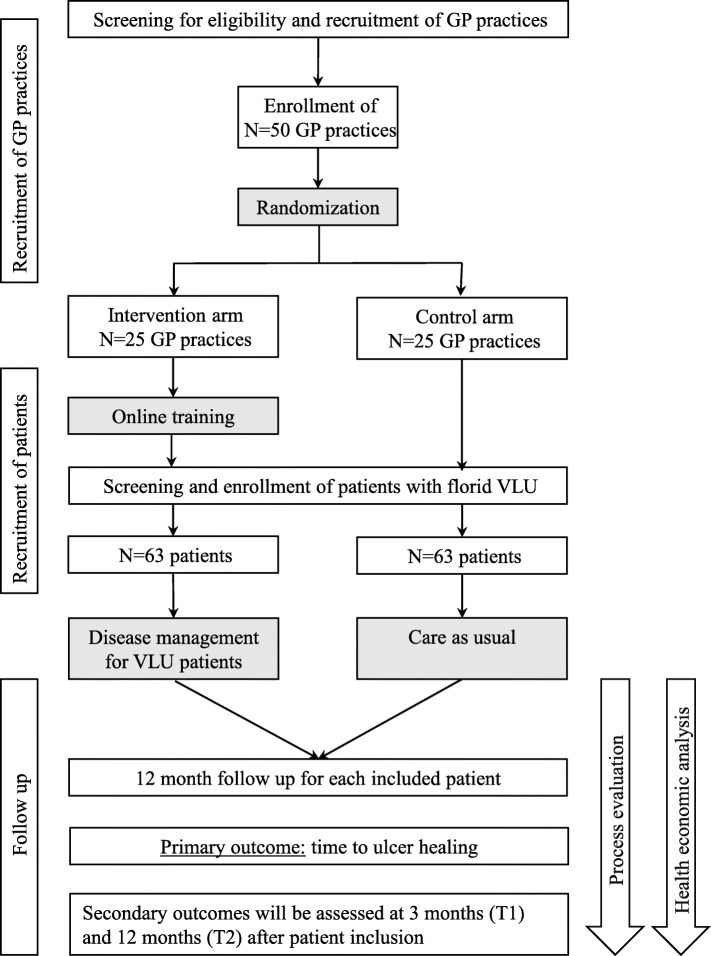
Trial scheme of the UCC trial

The UCC trial is a prospective cluster-randomized controlled multicenter trial designed to evaluate the intervention “Ulcus Cruris Care.” Fifty GP practices in the federal state of Baden-Wuerttemberg are intended to be recruited by the study center, the Department of General Practice and Health Services Research (University Hospital Heidelberg, Germany), and randomized 1:1 to intervention or control arm. Patients with VLU will be recruited by the participating GP practices, to include a total of 63 patients in the intervention arm and 63 patients in the control arm. The study hypothesis is that implementation of disease management for VLU patients in GP practices according to the “Ulcus Cruris Care” intervention can lead to faster wound healing for affected patients. The trial is accompanied by a health economic analysis and a process evaluation to assess the cost-effectiveness and applicability of the intervention. The SPIRIT reporting guidelines were followed for the preparation of the study protocol (see Additional file 1) [16]. Figure 1 offers an illustration of the trial scheme.

### Study population and eligibility criteria

Approximately 800 regional GP practices collaborating with the study center will be approached by information letters. Interested GP practices will be screened for inclusion by investigators from the study center according to eligibility criteria for GP practices shown in Table 1.

**Table 1:** Eligibility criteria for GP practices

Inclusion criteria	Exclusion criteria
• At least one medical assistant is routinely involved in chronic wound treatment and care	• No involvement of medical assistants in chronic wound treatment and care
• IT requirements for using the CareCockpit software met	• GP practices with more than 20 VLU patients per year
	• IT requirements for the use of the CareCockpit software not met
	• no declaration of consent given

*VLU* venous leg ulcer

Eligible physicians and medical assistants will be introduced to the trial via an information sheet and telephone contact for informed consent. Included GP practices are intended to recruit patients with at least one florid venous leg ulcer. Screening and review of patient eligibility will be performed by the responsible primary care physicians of included GP practices. Patient eligibility criteria are shown in Table 2.

**Table 2:** Eligibility criteria for patients

Inclusion criteria	Exclusion criteria
• Patient with VLU or mixed ulcer of predominantly venous origin	• Florid leg ulceration of other origins
• Ulcer duration ≤ 6 months	• Ulcer duration > 6 months (182 days)
• Age ≥ 18 years	• Ankle Brachial Index of affected lower extremity < 0.5 or ankle artery pressure < 60 mmHg
	• Decompensated heart failure (≥NYHA III)
	• Immobility
	• Age < 18 years
	• No ability to give consent

*VLU* venous leg ulcer

### Recruitment and trial timeline

Recruitment of 50 GP practices is planned to be accomplished by the end of the 2nd quarter of 2022. All included practices will screen patients likely to meet eligibility criteria and may include a maximum of up to 5 patients. Patients will be directly informed for consent by participating physicians and a patient information sheet. There will be a financial compensation for participants (GP practices and patients) for each study visit and an additional disbursement for complete data collection. Patient recruitment will be monitored by the study center and stopped as soon as the recruitment target of 63 patients is reached in each study arm. The trial timeline is shown in a SPIRIT figure (Additional file 2).

### Randomization

Included GP practices will be allocated in a 1:1 ratio to either intervention or control arm via cluster-randomization with variable block length. Randomization sequence will be generated using SAS software and is only accessible to the responsible study biometrician of the Institute of Medical Biometry, University Hospital Heidelberg, Germany.

### Intervention

Ulcus Cruris Care is a multifaceted intervention aiming to implement a disease management concept for outpatient treatment of VLU patients in GP practices. The intervention comprises four major components shown in Table 3.

**Table 3:** The intervention components in “Ulcus Cruris Care”

Components of the intervention “Ulcus Cruris Care”	
1	Online training and e-learning courses for GP practices
2	Standard operating procedures for VLU treatment
3	Software support for VLU disease management
4	E-learning courses and printable information for patient education

*VLU* venous leg ulcer

Participating GPs and medical assistants will receive joint online training via webinar according to a synchronous concept comprising 2 teaching units of 45 min each. In addition, asynchronous target-group-specific e-learning courses are provided via an online platform (https://welearn.academy). Online training and e-learning are mandatory to be completed before inclusion of the first patient and will be tracked via documentation and within the online platform. The didactic interventions are designed to support the implementation of a standardized disease management process for VLU patients in GP practices. Under GP supervision, medical assistants are intended to take a central role in wound treatment and patient monitoring and education. Central learning content comprises wound assessment, compression therapy, local wound therapy, and patient education. As a secondary learning objective, practical skills of correctly applying and assessing a compression bandage are to be conveyed. An online video demonstrating the application of compression bandages is integrated in the e-learning course, which is online available at any time. Online training and e-learning courses were designed and reviewed by physicians from the department for general practice and the department for dermatology at the University Hospital of Heidelberg.

To support standardized treatment, the practices are provided with standard operating procedures based on current guidelines for medical compression therapy, wound cleaning and local wound treatment for VLU.

A software support for wound documentation and patient monitoring was developed by the Department of General Practice and Health Services Research and is integrated into the case management software *care cockpit,* which has been developed for routine use in German GP practices [17]. Main function of the software module is a standardized monitoring of compression therapy, wound status, and patient education. Treatment success and wound healing can be checked via a progress overview. In addition, the creation and adaptation of a standardized treatment plan and scheduling of monitoring visits and recall is supported.

Patients are given access to a plain-language e-learning course and print-based information (asynchronous concept). The main content of the patient education comprises an introduction to VLU disease, compression therapy, local wound treatment, exercises, and general measures such as skin care and nutrition and are intended to support informed and active participation in the treatment process for patients and relatives. A video demonstrating application of compression bandages and step-by-step instructions for wound dressing changes and exercises are integrated in the e-learning course and are online available at any time. Patient education will be addressed in a standardized manner within monitoring visits in order to give regular opportunities to clarify open questions and promote active participation in the treatment process.

All intervention components are will be implemented on the practice level and are intended to support the treatment of patients with VLU in GP practices. Treatment and patient education fall under full responsibility of the treating physicians of the participating GP practices. Consequently, no unintended adverse effects are to be expected from the interventions.

### Control

No intervention will be performed in GP practices randomized to the control arm and included patients will receive usual care.

### Allocation concealment and blinding

Due to the character of the intervention, blinding of GP practices and patients is not feasible. Participating patients will not actively be informed about the allocation of their treating GP practice. However, it may be recognized if patients receive printable information material or access to patient e-learning as a part of the intervention. Investigators analyzing the primary outcome are blinded to group allocation.

#### Primary outcome

The primary outcome is the time from baseline to ulcer healing defined as complete re-epithelialization with no scab remaining. In case of multiple ulcers, all venous leg ulcers of both legs must meet the criteria of ulcer healing. Assessment of ulcer healing will be performed during regular visits in treating GP practices. If the criteria for ulcer healing are met according to judgment of treating physicians, pseudonymized photo documentation of the same day will be transferred to the study center. The photo documentation must comprise both legs in front and back view as well as close-ups of all leg ulcers. Two independent and blinded investigators who are qualified GPs will review the photo documentation to assess ulcer healing. In case of dissent, a third blinded investigator will be consulted for settlement. If ulcer healing is verified by two investigators, the date of the photo documentation will be recorded as the date of ulcer healing. If healing cannot be verified, treating physicians are informed and the verification process will be repeated weekly for 4 weeks. If the ulcer healing remains unaccomplished, initiation of the verification process will be repeated upon reassessment of the treating physician.

#### Secondary outcomes

Baseline parameters and the secondary outcomes are determined at baseline (T0), 3 (T1), and 12 months (T2) after patient inclusion (Table 4).

**Table 4:** Secondary outcomes

***Outcome***	***Time of measurement***
Number of patients with complete ulcer healing	T1, T2
Number, size of ulcers [cm^2^]	T0, T1, T2
Ulcer recurrence	T1, T2
Pain according to visual analog scale	T0, T1, T2
Health-related quality of life, assessed using the validated questionnaire (EQ-5D-5L)	T0, T1, T2
Depressiveness according to the validated Patient Health Questionnaire (PHQ-9)	T0, T1, T2
Patient satisfaction and information, assessed using the “Patient Assessment of Chronic Illness Care” (PACIC-5A)	T0, T1, T2
Adherence to venous leg ulcer treatment	T1, T2

T0 = baseline, T1 =3 months after inclusion, T2 = 12 months after inclusion

The number of patients with complete ulcer healing will be assessed according to the criteria defined for the primary outcome. Furthermore, the number and sizes of VLU will be assessed. Wound size is determined by the perpendicular method measuring the greatest length and greatest width in cm in perpendicular axes at time points T0, T1, and T2. Ulcer recurrence is defined as the occurrence of any new venous leg ulcer during the observation time. Pain intensity will be measured using the visual analog scale. To assess health-related quality of life, the validated EQ-5D-5L questionnaire of EuroQol Group will be used. Depressiveness will be assessed using the validated Patient Health Questionnaire (PHQ-9) [18]. The Patient Assessment of Chronic Illness Care (PACIC-5A) is used to survey patient satisfaction and quality of patient education [19]. Adherence to VLU treatment elements is assessed by the patient and by caregivers using a Likert scale of 1–5 (non-adherent to adherent) for attendance of appointments and implementation of caregiver recommendations for local wound treatment, change of wound dressings, and general measures. In order to identify barriers to adherence, a free text field is provided to facilitate an indication of individual problems with therapy elements.

#### Health economic analysis

A health economic analysis is performed to determine the efficiency of the intervention. To this end, cost-effectiveness analyses and cost-utility analyses are conducted. The output of the health economic evaluation is the total and incremental costs and effects (time to heal, health-related quality of life, other effects) of the intervention and control group. Using an incremental cost-effectiveness ratio (ICER), the incremental costs and effects are weighed against one another to quantify the cost-effectiveness of the intervention in comparison to treatment as usual. Only costs directly attributable to the treatment of the VLU are counted in the analysis.

In line with the IQWIG’s guidelines (CITE), the health economic analysis is conducted from the perspective of German statutory health insurances and their insured persons, meaning that only costs and effects incurred on the side of the health insurances and the patients are accounted for in the analyses [20]. Resource consumption and costs of inpatient care and outpatient care, drug prescriptions, remedies, and medical aids attributable to the treatment of VLU are counted in the analyses. The number of ambulatory care visits (surgery, dermatology, internal medicine, other specialist care), number of hospitalizations and days spent in inpatient care, days on sick leave, and travel costs will be collected using a healthcare utilization questionnaire. The medical care delivered or prescribed for participating patients will be collected using practice internal documentation. Here, the number of GP visits for wound treatment, prescribed home care and long-term care, prescribed remedies, and medical aids related to the VLU, as well as drug prescriptions and wound dressings will be recorded. The time horizon of the analysis is 12 months beginning from baseline (T0). Effects are measured at three points in time for both the intervention and the control group (T0, T1, T2), while costs are measured at one point in time, retrospectively at the end of each participant’s study trajectory (T2).

#### Process evaluation

The process evaluation of the UCC trial aims to explore factors contributing to or hindering successful implementation of the “Ulcus Cruris Care” intervention and its working mechanisms using a mixed-methods approach. At the beginning of the trial, the participating healthcare providers of both study arms will be surveyed by a questionnaire to collect demographics and descriptive data on the GP practices. A knowledge check of all participating caregivers will be performed using a standardized multiple-choice quiz comprising a total of 10 questions on disease, pathophysiology, diagnostics, and treatment of venous leg ulcers. At the end of the intervention period in the trial, a process evaluation questionnaire using decision questions, 5-point Likert scales, and free-text responses will be addressed to all participating caregivers and patients of the intervention arm to assess intervention fidelity, reach among patients, acceptance of intervention components, implementation effort, and potential sustainability and transferability to other settings. In addition, semi-structured interviews of a total of 35 persons are planned, including 10 GPs, 10 medical assistants, and 10 patients in the intervention arm as well as 5 regional stakeholders from health insurance and professional associations. Interviews will be conducted once practices have gained initial experience with the intervention and after completion of practice training and treatment of at least 2 patients within the study. Evaluation of the interviews and free-text answers from the questionnaires will be carried out in an inductive-deductive procedure using a framework analysis according to Gale et al. [21]. The Consolidated Framework for Implementation Research (CFIR) will be applied to categorize influencing factors during implementation [22]. Data will be organized and analyzed using the MAXQDA software.

### Data management

At each study visit, data will be recorded in an electronic case report form (eCRF) by the designated representative of the respective participating GP practice. Data entry will be performed as soon as possible after data retrieval and an explanation must be provided for all missing data. After completion of a study visit, a pseudonymized eCRF will be transferred to the study center. Pseudonymization is performed according to a software algorithm to assure that data may only be assigned to personal identity at the level of GP practices. Pseudonymized data of the health economic analysis will be transferred from the study center to the Institute for Applied Quality Promotion and Research in Health Care (Göttingen, Germany) for evaluation. All transferred data will be archived by the study center at the end of the trial. No formal data monitoring committee is planned, since no harms are to be expected by implementation of the UCC intervention. A trial steering group from the study center will meet at regular 2-week intervals to monitor trial conduct and data acquisition. To facilitate day-to-day support for participating GP practices a telephone hotline is provided the study center.

### Statistical methods

#### Sample size

The sample size calculation is based on the primary outcome “time to complete wound healing (“time-to-heal”)”. Assumptions are based on the literature: a healing rate after 12 weeks of 60% is assumed in the standard care (control group) [23] and, based on clinical studies on patient-centered interventions, of 80% [24] in the intervention group. With a significance level of *α* = 0.05, an assumed drop-out rate of 20%, and the assumption of exponentially distributed healing curves, a sample size of *n* = 55 patients per study arm is needed to achieve a power of 80% when applying a log-rank test in the classic two-group comparison. In order to take the cluster structure of the data into account (patients in doctors’ practices), the sample size is further adjusted by a design effect of 1.1, which is calculated from a cluster size of *n* = 3 patients per practice and a conservative intra-cluster correlation coefficient (ICC) of 0.05. This results in a total sample size to be recruited for the study of *n* = 126 patients (*n* = 63 per group) in *n* = 42 practices (*n* = 21 per group). In addition, in order to prevent possible drop-outs or recruitment bottlenecks among the GP practices, the target number of practices to be recruited is increased to *n* = 50 (*n* = 25 per group). This will increase the power in the study, as a larger number of clusters has a positive effect on the design effect. The sample size planning was carried out using PASS software.

#### Statistical analysis

The statistical analysis is carried out using a validated R environment with software version ≥ 4.0. The analysis of the quantitative data is performed according to scientific standards. The description of the baseline data will use appropriate descriptive measures such as mean, standard deviation, minimum, median, interquartile range and maximum for continuous parameters and scores, and using absolute and relative frequencies for categorical data. Homogeneity of the study arms will be evaluated by *t* test and chi-square test as appropriate.

The primary outcome “time to ulcer healing” is analyzed using a Cox proportional hazards regression model (shared frailty model) at a two-sided significance level of *α* = 5%. The variables “group,” “ulcer size,” “duration of VLU,” and “BMI” are included as fixed effects and the variable “practice” as a random effect. The use of this model generally increases the power compared to an ordinary log-rank test. Furthermore, the effect size is given by means of a point estimator (hazard ratio and rate difference at 12 weeks) with an associated 95% confidence interval. Further sensitivity analyses include the consideration of the cluster structure via averaging (per practice) and further regression models.

A non-inferiority analysis will be applied to the cost data, in order to determine whether the costs of care incurred in by the intervention group can be considered as high as or lower than the costs of care incurred in by the control group. The ICER will be calculated on the basis of the costs and effects of the intervention and control group as follows:

$$ \mathrm{ICER}=\frac{\varnothing \mathrm{costs}\ \mathrm{of}\ \mathrm{care}\ \mathrm{intervention}\ \mathrm{group}-\varnothing \mathrm{costs}\ \mathrm{of}\ \mathrm{care}\ \mathrm{control}\ \mathrm{group}}{\varnothing \mathrm{effects}\ \mathrm{intervention}\ \mathrm{group}-\varnothing \mathrm{effects}\ \mathrm{control}\ \mathrm{group}} $$

A formula based on the Fieller’s theorem will be used to determine the 95% confidence interval (CI) of the ICER. Using the 95% CI, the cost-effectiveness of the intervention will be interpreted in light of several realistic cost-effectiveness thresholds. Costs and effects are discounted at a rate of 3% p.a. Moreover, discounting rates of 0% and 5% are applied in a sensitivity analysis. The cost-effectiveness may differ for various subgroups and variables unaffected by the intervention. These should be compared or controlled for statistically. To account for uncertainty in the measured parameters, both deterministic and probabilistic sensitivity analyses will be performed, where variables affecting the cost-effectiveness are randomly drawn from a distribution informed by observed data and academic literature.

The evaluation of further secondary outcomes will be carried out with appropriate descriptive measures and comparisons of the study groups using t-tests for continuous variables and chi-square tests for categorical variables. The corresponding effects are described using point estimators with associated 95% confidence intervals. The relationship between variables is checked using the Spearman correlation coefficient. Wherever appropriate, statistical graphs will be provided to visualize the results. Details of the statistical analysis will be further specified in a statistical analysis plan, which will be completed before database closure.

### Ethical approval

The ethics committee of the University of Heidelberg reviewed and approved this study on August 5_,_ 2021 (reference number: S-608/2021, see Additional file 3). Written, informed consent will be obtained from all participants and documented by consent forms, which can be provided upon request. Protocol modifications are not foreseen and have to be approved by the ethics committee. In case of modification, study participants will be sent the new protocol version and informed via e-mail and phone calls.

### Good Clinical Practice

The trial is conceived and will be conducted according to all relevant national and international rules and regulations (ICH-GCP, Declaration of Helsinki 2013).

### Registration

The study protocol has been registered with the German Clinical Trials Register (http://www.drks.de/) on August 30, 2021, under the registration number DRKS00026126. In case of modifications to the protocol, the register record will be updated.

## Discussion

The UCC trial is the first study to evaluate a disease management concept for the treatment of VLU. The Ulcus Cruris Care program was developed to support standardized evidence-based and patient-centered treatment of VLU in GP practices. Its intervention elements are intended to enhance the knowledge and practical skills of caregivers, support standardized treatment of VLU, and promote informed and active participation of patients. As an inherent objective, Ulcus Cruris Care is intended to promote the application of compression therapy by supporting knowledge on the caregiver side and patient adherence, which are the main barriers to its use [13, 15, 23].

With regard to limitations, it has to be mentioned that allocation concealment for participating patients is not feasible. Patients will not be informed about the allocation of their treating GP practice; however, patients in the intervention group receive access for e-learning and print-based information. Furthermore, patient recruitment may be affected by the ongoing COVID-19 pandemic. However, to ensure its feasibility, timelines and recruitment aims have been adapted according to our experiences in currently ongoing studies.

The UCC trial will help to evaluate whether the Ulcus Cruris Care intervention may lead to faster wound healing, a higher health-related quality of life and a lower use of medical resources. If the intervention turns out to have a positive impact on assessed outcomes, comprehensive implementation in primary care may be considered. Furthermore, if successful, the concept of Ulcus Cruris Care may serve as a role model for further disease management concepts for chronic wound treatment.

## Trial status

The trial will be conducted according to the presented protocol (version 1 as of July 19, 2021). Recruitment of GP practices will begin in the 4th quarter of 2021 and is planned to be finished until the end of 2nd quarter of 2022. Patient recruitment is scheduled for the 12-month period of 2022. The results of the UCC trial are expected for the 1st quarter of 2024 and will be communicated via publications, the website of the trial (https://ulcuscruris.care), and the German clinical Trials Register.

## Supplementary Information

**Additional file 1.** SPIRIT checklist of the UCC trial.docx

**Additional file 2.** SPIRIT figure of the UCC trial.docx

**Additional file 3.** English confirmation ethics approval UCC trial.pdf

**Additional file 4.** Translation of the original funding documentation.pdf

## Data Availability

All transferred data will be archived by the study center at the end of the trial. Public access to the full protocol, participant-level dataset, or statistical code is not planned. However, the full version of the study protocol as well as anonymized trial data will be available upon reasonable request.

## Abbreviations

GP: General practitioner
VLU: Venous leg ulcer
