# Supplementary material for: Primary care disease management for venous leg ulceration—study protocol for the Ulcus Cruris Care [UCC] randomized controlled trial (DRKS00026126)
Source: Trials. 2022 Jan 20;23:60. doi: 10.1186/s13063-021-05944-9 (PMC8771170; doi:10.1186/s13063-021-05944-9)
Supplement: Supplementary file 2 — Additional file 2. SPIRIT figure of the UCC trial.docx [file 13063_2021_5944_MOESM2_ESM.docx]

# SPIRIT Figure of the UCC trial

| **Study preparation**  (Q4 2021 – Q2 2022) | | | | **Study period**  (Q1 2022-Q4 2023) | | | |
| --- | --- | --- | --- | --- | --- | --- | --- |
|  | **Recruitment of GP practices** | **Allocation** | **Patient recruitment*** | **Assessment visit****  (Baseline) | **Study visit 1****  (3 months) | **Study visit 2****  (12 months) | **End of trial** |
| **Time point** | 4^th^ quarter 2021 | Before T_0_ | Before T_0_ | T_0_ | T_1_ | T_2_ | 1^st^ quarter 2024 |
|  | | | | | | | |
| **Recruitment** | **x** |  | **x** |  |  |  |  |
| **Informed consent** | **x** |  | **x** |  |  |  |  |
| **Enrolment** | **x** |  | **x** |  |  |  |  |
| **Allocation** |  | **x** |  |  |  |  |  |
| **Data collection** |  |  |  | **x** | **x** | **x** |  |
| **Data analysis** |  |  |  |  |  |  | **x** |
| **Interventions** | | | | | | | |
| **Online training***** |  | **x** | **x** |  |  |  |  |
| **E-Learning***** |  | **x** | **x** |  |  |  |  |
| **Standard operating procedures***** |  |  |  |  |  |  |  |
| **Software support***** |  |  |  |  |  |  |  |
| **Patient information & E-Learning***** |  |  |  |  |  |  |  |
| **Control group**  no intervention |  |  |  |  |  |  |  |
| **Assessments** | | | | | | | |
| **Baseline variables** |  |  |  | **x** |  |  |  |
| **Primary outcome**  (Time-to-heal)**** |  |  |  |  |  |  |  |
| **Secondary outcomes** (for a list of variables see manuscript) |  |  |  |  | **x** | **x** |  |

GP = general practitioner

* Patients are recruited by participating GP practices.

** The study visits timeline refers to the trial timeline of a single study participant (patient).

*** Interventions will be implemented in general practices of the intervention group only. Webinar and E-Learning need to be finished before patient recruitment. The E-Learning remains available for the duration of the trial.

****Participating general practitioners are instructed to report wound healing at any time throughout the trial.
