# Supplementary material for: Primary care disease management for venous leg ulceration—study protocol for the Ulcus Cruris Care [UCC] randomized controlled trial (DRKS00026126)
Source: Trials. 2022 Jan 20;23:60. doi: 10.1186/s13063-021-05944-9 (PMC8771170; doi:10.1186/s13063-021-05944-9)
Supplement: Supplementary file 3 — Additional file 3. English confirmation ethics approval UCC trial.pdf [file 13063_2021_5944_MOESM3_ESM.pdf]

MEDIZINISCHE
FAKULTÄT
HEIDELBERG

Ethikkommission der Med. Fak. HD | Alte Glockengießerei 11/1 | 69115 Heidelberg

Herrn Prof. Dr. med. Dipl. Soz. Joachim Szecsenyi
Universitätsklinikum Heidelberg
Abt. Allgemeinmedizin und Versorgungsforschung
Im Neuenheimer Feld 130.3
Marsilius Arkaden, Turm West
69120 Heidelberg

**Universität Heidelberg
Ethikkommission der
Med. Fakultät**

Alte Glockengießerei 11/1
69115 Heidelberg

Tel. +49 6221 56264-60 (Zentrale)
Fax +49 6221 56264-80
ethikkommission-I@med.uni-heidelberg.de

www.medizinische-fakultaet-hd.uni-heidelberg.de/ethikkommission

Vorsitz:
Prof. Dr. med. Dr. h.c. Thomas Strowitzki

Stellv. Vorsitz:
Prof. Dr. med. Klaus Herfarth

Geschäftsleitung:
Dr. med. Verena Pfeilschifter

27.08.2021
-si

CONFIRMATION

Reference No: **S-608/2021**

Title: **Cluster-randomisierte kontrollierte Interventionsstudie zur Evaluation der Implementierung eines evidenz-basierten und patientenorientierten Versorgungskonzepts für die Behandlung des Ulcus cruris venosum in der Hausarztpraxis („Ulcus Cruris Care“)**

Dear Professor Szecsenyi,

We hereby confirm that the above-mentioned research project has been reviewed and approved by the Ethics Committee of the Medical Faculty of the University of Heidelberg. The approval of the Ethics Committee (Berufsrechtliche Beratung), dated to 05.08.2021, has been sent to you on 05.08.2021.

Kind regards

Digital unterschrieben von Sylke Siebrecht
Datum: 2021.08.27 11:38:44 +02'00'

Dr. rer. nat. Sylke Siebrecht
Sonstige Studien

399893 QM 15
ISO 9001:2015
TÜV SÜD ISO 27001

BIC SOLADEST600
IBAN DE64 6005 0101 7421 5004 29

.
