# Supplementary material for: Primary care disease management for venous leg ulceration—study protocol for the Ulcus Cruris Care [UCC] randomized controlled trial (DRKS00026126)
Source: Trials. 2022 Jan 20;23:60. doi: 10.1186/s13063-021-05944-9 (PMC8771170; doi:10.1186/s13063-021-05944-9)
Supplement: Supplementary file 4 — Additional file 4. Translation of the original funding documentation.pdf [file 13063_2021_5944_MOESM4_ESM.pdf]

Gemeinsamer
Bundesausschuss
Innovationsausschuss

Geschäftsstelle

Besuchsadresse:
Gutenbergstraße 13
10587 Berlin

Ansprechpartner/in:
Dr. Annelie Brendel

E-Mail:
Annelie.brendel@if.g-ba.de

Internet:
www.if.g-ba.de

Unser Zeichen:
01VSF19043

Datum:
1. September 2021

Innovationsausschuss beim G-BA, Postfach 12 06 06, 10596 Berlin

Prof. Dr. Joachim Szecsenyi
Universitätsklinikum Heidelberg
Abteilung Allgemeinmedizin und
Versorgungsforschung
Im Neuenheimer Feld 130.3
69120 Heidelberg

Confirmation government funding and assessment corresponding to a peer-review

Dear Professor Szecsenyi,

we hereby confirm that the project „ULCUS CRURIS CARE – Etablierung eines patientenorientierten und evidenzbasierten Versorgungskonzeptes für die Therapie des Ulcus cruris venosum in der Hausarztpraxis“ is funded by the German Innovation Fund with the funding number: 01VSF19043.

Projects funded by the German Innovation Fund run through an assessment procedure that correspond to a peer-review.

Details of the assessment procedere are described in § 9 of the Code of procedure of the council of the German Innovation Fund: https://innovationsfonds.g-ba.de/downloads/media/6/Verfahrensordnung_Innovationsausschuss_2015-12-14.pdf

The application for „Ulcus Cruris Care“ was assessed by an council of experts and finally (under consideration of the results of the experts) of the council of the German Innovation Fund (both councils existing of 10 members).

Gemeinsamer Bundesausschuss
Innovationsausschuss

The council of the German Innovation Fund is located at the Federal Joint Committee (G-BA)[1]. The Federal Joint Committee is the highest decision-making body of the joint self-government of physicians, dentists, hospitals and health insurance funds in Germany. It issues directives for the benefit catalogue of the statutory health insurance funds (GKV) for more than 73 million insured persons and thus specifies which services in medical care reimbursed by the GKV.

The documentation of the Ulcus Cruris Care project is accessible on the website of the funding body:
ULCUS CRURIS CARE – Etablierung eines patientenorientierten und evidenzbasierten Versorgungskonzeptes für die Therapie des Ulcus cruris venosum in der Hausarztpraxis - G-BA Innovationsfonds

Please contact us if additional questions arise.

Sincerely yours,

Brendel

Dr. Annelie Brendel

[1] in German: Innovationsausschuss beim Gemeinsamen Bundesausschuss.
